# Multidirectional Polymer Waveguide Lattices for Enhanced Ultrawide-Angle Light Capture in Silicon Solar Cells

**DOI:** 10.1021/acsaem.2c01630

**Published:** 2022-07-22

**Authors:** Nannan Ding, Ian D. Hosein

**Affiliations:** Department of Biomedical and Chemical Engineering, Syracuse University, Syracuse, New York 13244, United States

**Keywords:** polymers, photopolymerization, coatings, solar cells, energy, optics, waveguides

## Abstract

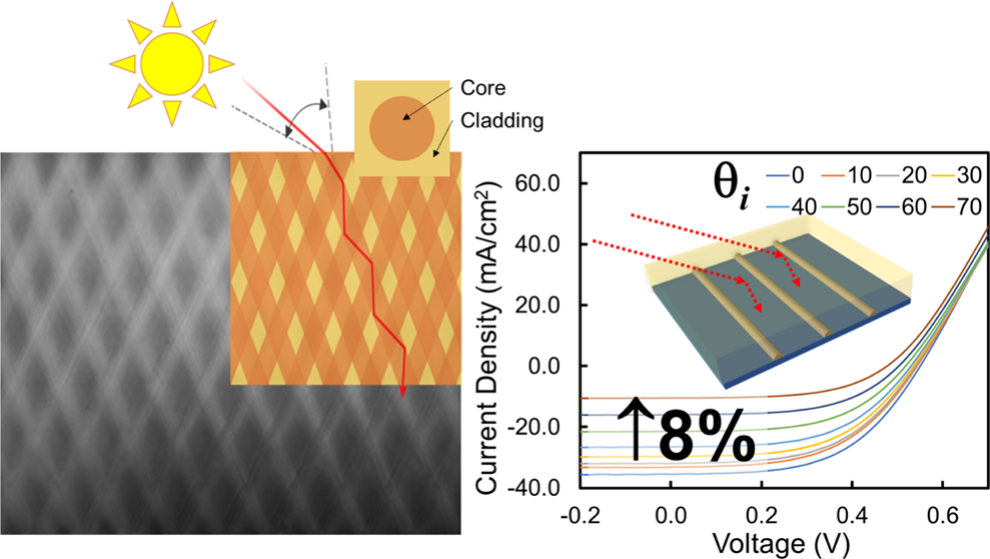

We report the synthesis and characterization of a polymer thin-film structure consisting of two intersecting broadband optical waveguide lattices, and its performance in wide-angle optical energy collection and conversion in silicon solar cells. The structures are synthetically organized via the concurrent irradiation of photoreactive polymer blends by two arrays of intersecting, microscale optical beams transmitted through the medium. Through optical beam-induced photopolymerization and photopolymerization-induced phase separation, well-organized lattices are produced comprising of cylindrical core–cladding waveguide architectures that intersect one another. The optical waveguide properties of the lattices transform the transmission characteristics of the polymer film so that incident optical energy is collected and transmitted along the waveguide axes, rather than their natural directions dictated by refraction, thereby creating efficient light-collecting capability. The embedded structures collectively impart their wide-angle acceptance ranges to enable the film to efficiently collect and interact with light over a large angular range (±70°). When employed as the encapsulant material for a commercial silicon solar cell, the novel light collection and transmission properties result in greater wide-angle conversion efficiency and electrical current density, compared to a single vertically aligned waveguide array. The sustained and greater conversion of light afforded by the encapsulating optical material promises to increase solar cell performance by enabling ultrawide-angle solar energy conversion.

## Introduction

Solar cells continue to play an important role in sustainable, renewable energy conversion to support a green, cleaner energy grid. Toward advancing solar cell technologies, increasing the total solar energy captured and converted into electricity is an attractive option. One approach is to deploy light management and light collection strategies, to increase total light capture and mitigate potential losses, thereby maximizing conversion and electrical output. From an optics perspective, this can be achieved using new types of dielectric structures that elicit novel or stronger light–matter interactions via such effects as antireflection, optical mode coupling, geometric refractive optics, plasmonics, and even ray-steering. Such effects have been realized using optical material structures such as nanoparticle surface coatings,^1^ nanostructured diffractive, diffuse and scattering layers,^2−5^ gratings,^6^ nanotexturing,^7^ geometric optical structures,^3,8^ one-dimensional (1D) and two-dimensional (2D) lens arrays,^9−11^ microscale patterning,^12^ or even more sophisticated methods such as contact cloaking,^13−16^ modified front-contact shape,^17^ and alternatively designing back-contact architectures^18^ and transparent electrodes.^19^

The above approaches have focused primarily on maximizing collection efficiencies and total current output particularly for direct solar irradiation, namely, light at or close to normal incidence. However, there remain significant opportunities in the development of optical structures that increase the capture of non-normally incident light. Commercial front-contact solar cells show drastically reduced conversion efficiency and output at increasingly greater incident angles (relative to the surface normal) owing in part to significant shading losses from the screen-printed metallic contacts in addition to increased reflection at the solar cell surface.^20^ Consequently, as the sun moves along its diurnal trajectory, both over the course of a day and across seasons, solar irradiation at more glancing angles yields reduced conversion efficiency owing to these shading effects and nonoptimal ray propagation. Additionally, owing to geographic or infrastructure restraints, solar panel installations may be placed in nonoptimal locations (e.g., ill-aligned, slanted rooftops, city locations, etc.) or even around transportation infrastructure, consequently necessitating further the need for better “omni-directional” collection of solar irradiation. Hence, there remain opportunities to develop optical materials that help sustain and manage light collection over a greater range of incident angles, and, particularly, for wide incident angle radiation (i.e., >40°). Importantly, the loss in efficiency and performance at wider angles is so strong that even marginal increases in light capture and conversion can aid to increase overall solar cell output. Currently, wide-angle energy conversion in solar cells remains a relatively unexplored area of research, with only a few studies having examined possible performance enhancements through such methods as surface texturing, micro-lensing, and transparent electrodes.^9−12,19,21^

Employing optical waveguiding structures provides another straightforward approach to manage light transmission over a solar cell.^22^ This approach exploits the inherent angular acceptance range for incident light^23^ of a waveguide structure (particularly across the air–structure interface) and subsequent guided transmission along the waveguide’s long axis as a means to control the direction of light propagation. Creating waveguide structures in a planar film could then serve the dual objectives of both the encapsulation material and a means to alter and manage the transmission properties of light, providing more favorable, direct incident angles and greater transmission flux when light finally reaches the solar cell. We have investigated the fabrication of polymer films consisting of a single array of waveguide elements such as embedded air prisms,^13^ 1D waveguide slabs,^8^ vertically aligned microfiber arrays,^24−28^ and submillimeter cylindrical waveguides (WGs) in embedded matrices.^20,24,29,30^ All such structures provide some, but only a mild enhancement in light capture at greater incident angles compared to a uniform (i.e., nonstructured) encapsulating resin, but the concept nonetheless is promising. Specifically, arrays or even bundles of cylindrical waveguide architectures, namely, those with a high-index cylindrical core embedded in a low-index cladding, have provided many examples of their capability to collect and alter light transmission. The arrays of cylindrical waveguides can be produced via the irradiation of photopolymerizable media with an array of microscale optical beams, which undergo self-trapping and induce self-writing of the cylindrical core geometry along their length.^31,32^ These arrays show novel light collection and manipulation capabilities.^8,33,34^ We previously exploited the waveguide collection optics of cylindrical waveguides in a vertically aligned array (i.e., parallel to the surface normal) synthesized in photoreactive polymer blends.^20,24,30^ Through optimizations of array spacing (i.e., lattice period) and blend composition, the total light captured could be increased for incident angles up to 40°,^29^ but performance still drops dramatically for incident angles >40°.

The collection capabilities of optical waveguide structures, across an air–material interface, are limited by their acceptance “cone”, an interval of incident angles entering the waveguide core that will satisfy the total internal reflection requirement to enable optical waveguiding. The concept of “light collection” using a waveguide works by collecting light within this acceptance cone, followed by confinement inside the waveguides which restricts transmission along its axis across the material’s thickness. In the theoretical case of a vertically aligned waveguide embedded in a thin-film medium and having an acceptance range of ±θ, light would assume an effective transmitted direction of 0° in side the medium, reaching the solar cell at this effective angle on the other side of the film. The more light propagates at effectively normal incidence, the greater the boost to collection efficiency.^29^ The waveguide acceptance range (Δθ) for a cylindrical waveguide aligned with its axis normal to the air–material interface is calculated based on the refractive index of the waveguide core (*n*_1_) and the surroundings (i.e., cladding) (*n*_2_) according to .^23^ The acceptance cone is symmetric about the surface normal, and the width is dictated by the refractive index difference between the core and the cladding materials. Using the refractive index values of the polymers employed in our previous work (*n*_1_: 1.653, *n*_2_: 1.412),^29^ a maximum acceptance range of ±30° is possible. What this implies is that light incident within an angular range of ±30° can be collected by and guided along the waveguide axis to the other side of the material; however, beyond that angular range, the beneficial enhancement to collection efficiency drops significantly. Hence, appropriate variations in the structure are needed to enable collect at ultrawide angles (i.e., >40°), which are beyond the fundamental acceptance cone of vertically aligned waveguides.

One solution is to produce waveguides at slanted orientations in encapsulation materials so as to rotate the acceptance cone to collect light to more glancing incident angles. This approach has been considered with multiple-waveguide arrangements possessing different orientations to sweep the collection window over a greater angular range.^33−35^ Also, waveguides with monotonically varying radial orientations have also been shown suitable for beam steering.^34,35^ Multiwaveguide lattices have also been produced in single-component media with multidirectional transmission properties.^36−38^ Hence, the use of slant-oriented waveguide can provide the opportunity to suitably position the acceptance ranges of the waveguide arrays to enable the capture of light at even wider incident angles.

Here, we propose the synthesis and application of thin films comprising two symmetrically arranged intersecting waveguide lattices, oriented at non-normal incident angles relative to the air–film interface, to enable ultrawide-angle light capture and increased conversion in solar cells. In this work, the two intersecting waveguide lattices are prepared through photopolymerization of photoreactive polymer blends via irradiation with two arrays of microscale visible-light optical beams. The optical beams drive the polymerization of cylindrical channels along their propagation length^31^ and eventually cause spatially-controlled polymerization-induced phase separation (PIPS) of the blend.^39−41^ The optical beams become self-trapped in the polymerizing higher-index medium (both free-radical and cationic systems),^31,39,42^ preserving its collimated nature, in a process referred to as light-induced self-writing.^43^ Thereby, each cylindrical channel evolves into a high-index core and low-index cladding architecture, essentially developing a refractive index difference to function as optical waveguides. We have proposed their formation as well as correlated their structural quality to different blend and polymerization parameters in Multiphysics simulations.^44^ Unlike previous work which elicits the formation of such structures in single-component media, herein, the phase evolution of a binary blend of high- and low-index polymers enables such structures to achieve larger refractive index differences (∼Δ*n* of 0.01 vs 0.001), which significantly widens the waveguide’s acceptance range. Their slanted orientation rotates their acceptance ranges to more glancing angles, i.e., closer to the air–film interface, enabling ultrawide-angle incident light to be accepted and transmit along the waveguide axes. Their effective transmission in the material occurs at a smaller incident angle relative to their entry, thereby providing a more direct path toward the solar cell and mitigating any possible flux over top contacts. We have already demonstrated the capability to produce single vertically aligned arrays of cylindrical optical waveguides.^32^ Herein, this is the first demonstration of intersecting waveguides produced from polymer blends, revealing the advanced nature of the synthetic organization of the structures via photopolymerization and PIPS. We explore four different polymer blend formulations in terms of component weight fractions and photoinitiator concentration to reveal correlations between the polymer blend and photopolymerization conditions to the optical properties of the structures and performance of encapsulated solar cells. We show enhancements in both the conversion efficiency and electrical output as a function of incident angle relative to the comparative baseline of a single vertical array of waveguides. This work demonstrates a straightforward approach to produce complex optical waveguide structures with ultrawide-angle light collection capabilities toward their application in enhanced solar light capture and conversion in solar cells.

## Experimental Methods

### Materials

As the components of the photopolymerizable polymer blends, a high-refractive-index Norland Optical Adhesive 65 (NOA65) was purchased from Norland Products, Inc. and a low-refractive-index epoxide-terminated poly(dimethylsiloxane) (PDMS) oligomer from Gelest, and camphorquinone (CQ) free-radical photoinitiator from Sigma-Aldrich. The cationic initiator (4-octyloxyphenyl) phenyliodonium hexafluoroantimonate (OPPI) was purchased from Hampford Research, Inc. All chemicals were used without further purification.

### Mixture Preparation

Polymer blends with two different weight fractions (50/50, 20/80, wt %/wt % of total mixture, PDMS/NOA65) were explored herein, along with two different free-radical photoinitiator (CQ) concentrations (1.5 and 2.5 wt % of total mixture), respectively, and a corresponding OPPI of 2.5 wt % of the mixture. The photocurable formulations were prepared by thoroughly mixing NOA65, PDMS, CQ, and OPPI with their respective weight fractions in an aluminum-foil-wrapped vial. A magnetic stir bar was added to facilitate the formation of a homogeneous mixture under isolated conditions from ambient air and light. The mixture was stirred for 24 h before use. The mixture was injected into a Teflon ring cell (2 mm thickness) to its full volume for photopolymerization.

### Photopolymerization

A single chrome mask (Photosciences, Inc.) consisting of a square array of apertures of diameter 40 μm and spacing of 200 μm was used to generate the arrays of optical beams. Two collimated light-emitting diode (LED) light sources were aligned on an optical board on opposite sides of the mask and oriented to pass their light through the mask from below at the same incident angle, resulting in the symmetric formation and projection of two arrays of optical beams above the photomask. The orientation of the LEDs was ∼±45° with respect to the surface normal of the mask. The photocurable mixture in the ring cell was placed approximately over the center of the confocal region (i.e., the common region where the LED “spotlight” impinges on the mask). After placement, the mixture was then exposed to the collimated blue light (λ = 470 nm) generated from the LEDs (∼12–13 mW cm^–2^).

The irradiation procedure consisted first of alternating exposure of the mixture to one of the LEDs, followed by simultaneous irradiation. In more detail, the photopolymerizable mixture was alternately irradiated by each individual LED (with the other turned off) for a 5 min duration, and this process was repeated 10 times, resulting in a total of 50 min exposure of the mixture to each LED. This provided the opportunity for each individual array of optical beams to inscribe their initial structure unabated by the optical beams generated from the other LED. Subsequently, both LEDs were turned on simultaneously to irradiate the medium until the photopolymer blend was fully cured.

### Characterization

A Zeiss Axioscope equipped with an Axiocam 105 color camera operated by Zeiss imaging software was used to capture images of the cured polymer blends. All samples were imaged in reflection mode. The transverse spatial intensity profile of incandescent light from a QTH source transmitted through the materials was captured with a CCD camera, using an optics setup described previously.^31,33^

### Solar Cell Characterization

A planar multicrystalline silicon screen-printed solar cell (5 cm × 5 cm × 0.5 mm) with a measured short-circuit current density of 35.5 mA/cm^2^ was used in experiments. The photopolymerized samples were laminated on the solar cell (15 cm, 5 cm, 0.5 mm), which was first covered with a 0.12 mm layer of PDMS (Sylgard). External quantum efficiency (EQE) spectra and total external quantum efficiency were measured and calculated, respectively, as described previously.^29^ Angle-resolved measurements were performed as also described in our previous work.^30^ All measurements were conducted on the same region of the solar cell. Current density–voltage (*J*–*V*) curves of the encapsulated solar cell were collected under solar simulated irradiation (AM 1.5 G).^30^

## Results

**Figure 1:**
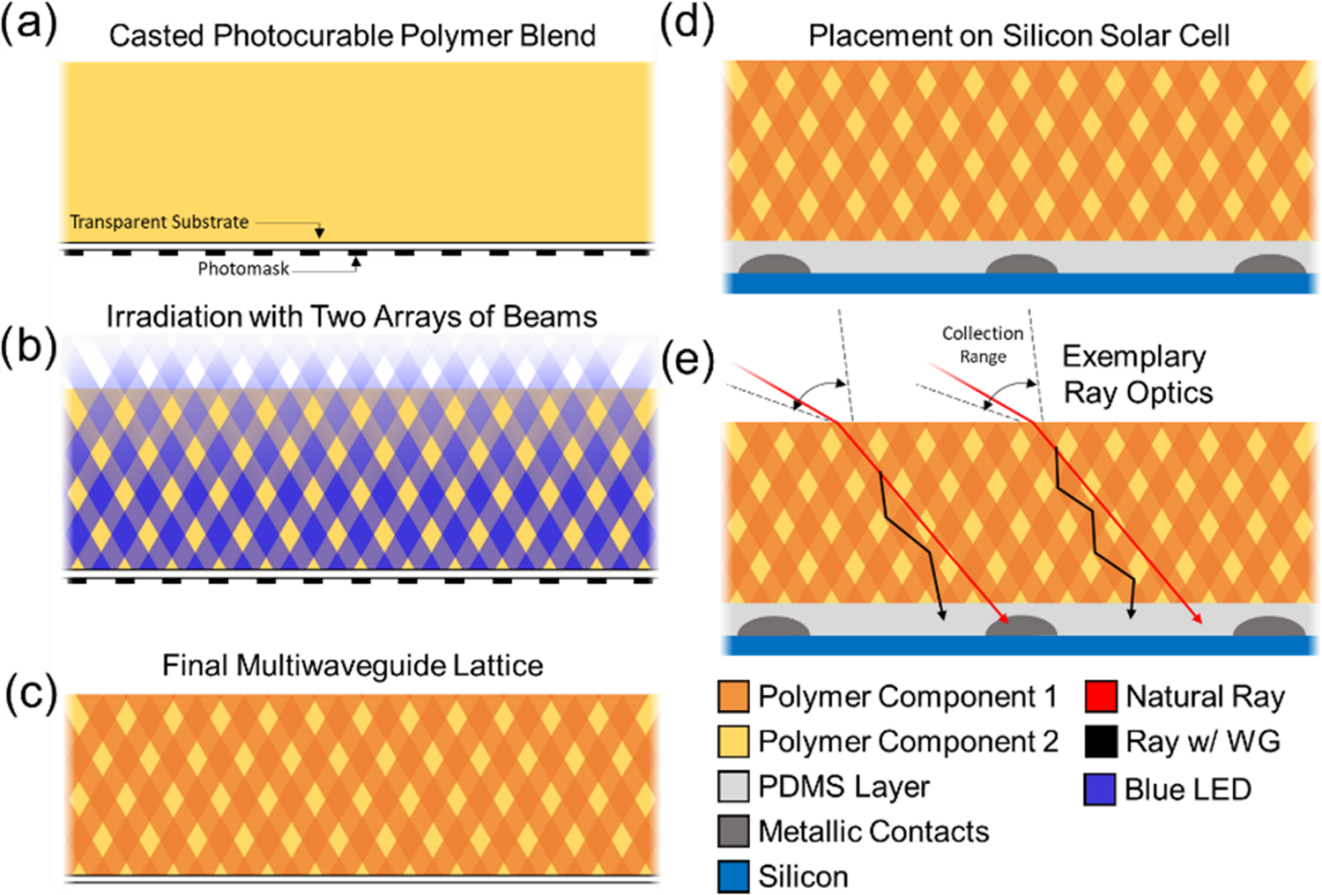
Schematic of the production of a multiwaveguide lattice structure, consisting of two symmetrically oriented waveguide arrays (±θ relative to the surface normal). The process for their fabrication is as follows: (a) a photopolymerizable blend is cast over a thin transparent (glass) substrate, which is positioned over an optical photomask pattern. (b) Two LED light sources are transmitted through the mask into the resin from the bottom, creating two arrays of intersecting optical beams. (c) Over the duration of light exposure, the blend evolves into its two intersecting waveguide (2W) array structure with a binary phase morphology consisting of the high-refractive-index polymer in the regions of irradiation and the low-refractive-index polymer in the dark regions (i.e., nonilluminated ). (d) Processed optical materials are placed over a silicon solar cell whose surface has been planarized with a thin protective PDMS layer. (e) Schematic of the operational principle of the waveguide lattice structure, whereby light (red rays) in a uniform medium that would hit a front contact is now accepted by the waveguide lattice structure (black rays) and transmitted along a more direct optical pathway toward the solar cell at a smaller incident angle than its initial one when refracted through the air–material interface.

Figure 1 illustrates the procedure for synthetically organizing two waveguide lattices, with symmetrically oriented directions (i.e., ±θ) with respect to the mask surface normal. A photocurable binary component resin is cast over a transparent substrate, which lies on a photomask (Figure 1a). LED illumination is directed through the photomask at a non-normal incident angle (Figure 1b). The mask transforms the initially uniform LED illumination into an array of microscale optical beams that propagate through the resin at a refracted angle. The individual beams in both arrays all induce the photopolymerization of cylindrical fibers aligned in the propagation direction of the beams (Figure 1c) and comprise predominately of polymerizing NOA65, owing to its faster free-radical polymerization. Over time, the PDMS polymer component is expelled, via PIPS,^32^ into the dark regions surrounding the propagating optical beams. Thereby, overall, the entire resin is organized into a structure possessing multiple (i.e., two) intersecting optical waveguide lattices. Polymer films of 2 mm thickness were selected owing to similar thicknesses with commercial encapsulations. The selected thickness is also sufficient to allow the formed cylindrical cores to be long enough to operate as waveguides (rather than just short cylinder dielectric structures) as well as for the film to be mechanically robust. The as-synthetized thin film is then placed on a front-contact solar cell (Figure 1d), which was first encapsulated with a very thin PDMS layer to planarize the surface and ensure intimate contact with the film. This process has the benefit of not requiring any structural changes to the native photovoltaic cell, and all processing is during the encapsulation stage. Ideally, any front-contact solar cell of any composition can be encapsulated, enabling the streamlining of the encapsulation procedure in photovoltaic panel manufacture.

Conceptually, in terms of the optics, unlike a single vertically aligned waveguide array for which the collection range is symmetric about the surface normal (±θ),^30^ in this multiwaveguide lattice structure, the collection ranges of the slant-oriented waveguides rotate clockwise and counterclockwise for waveguides with positive and negative angle orientations, relative to the surface normal (positive-wise in this case is clockwise rotation), as shown in Figure 1e. As schematically illustrated for optical rays at a large incident angle, in a uniform resin, such light rays (represented by red in Figure 1e) may be ill-fated to impinge on a contact, which can induce reflective losses, thereby reducing total light conversion. This is especially the case at large incident angles, where there is a greater fractional area of the solar cell screened by the contacts (via a line-of-sight principle), and hence more light is lost. However, using a polymer film composed of optical waveguides (WGs), those same light rays (now represented by black in Figure 1e) now can be collected by the individual cylindrical waveguides in an array. This effect of collection now allows light transmission along the waveguide core’s long axis, via optical waveguiding and total internal reflection effects indicated by the black rays bouncing off the waveguide core–cladding interface. Because the waveguides have a smaller angle than the initial incident angle of light, they provide a more direct pathway (i.e., smaller, less oblique effective incident angle) toward the active regions of the solar cell. As a result, by lowering the incident angle of incoming light rays, by now transmitting them along the waveguides, they will experience less fractional area of the screening top contacts (i.e., less top contact shading) at the solar cell surface. Hence, more incident light collected by the waveguides will be converted by solar cells (black rays) compared to a uniform coating (red rays). Shading losses are especially exacerbated at very high incident angles where there is more screening;^13^ hence, the waveguide lattice structure would prominently mitigate this effect, thereby enhancing total energy conversion at ultrawide angles and providing an overall very large angular range of operation (i.e., −70 to +70°).

**Figure 2:**
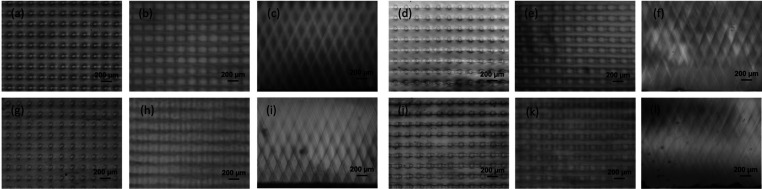
Optical microscopy images of the intersecting waveguide lattice structures. Structures are shown in three-series images of top, bottom, and cross-sectional views. Polymer blend parameters are: (a–c) 20/80 and 2.5 wt % CQ, (d–f) 20/80 and 1.5 wt % CQ, (g–i) 50/50 and 2.5 wt % CQ, and (j–l) 50/50 and 1.5 wt % CQ. Cross-sectional images are oriented right side up, with the LED light having propagated upward to synthesize the structures.

Figure 2 shows optical microscopy images of the cross sections of the multiwaveguide lattices produced with two different relative weight fractions of the polymer components and two different photoinitiator (i.e., CQ) concentrations. The measured angles of the waveguides were θ ≈ 25°, which is in accordance with the expected propagation angle of the optical beams when refracted into the polymer medium. Specifically, according to Snell’s law, a 45° incident angle leads to an ∼25° internal angle in an initially homogeneous resin with average refractive indices of ∼1.6 and ∼1.5 for 20/80 and 50/50 blends, respectively. The intersecting nature of the two arrays (orientations at approximately −25 and +25°) is clearly revealed in the optical microscopy images. The core clad architecture is visible in both the top and bottom images of the multidirectional waveguide arrays produced from both 50/50 and 20/80 blends (Figure 2a,c); however, the circular cross-section is clearer for 20/80 blends. Likewise, the cross-sectional images reveal that the waveguide cylinders become splayed with streak-like features or more divergent further along their cylindrical axes, to the extent that they are not as easily distinguishable at the end of the film. However, the waveguide cylindrical geometry is quite easily delineated over the depth of the sample for the 20/80 blends, although broadening of the diameter is still observed. With regard to CQ content, higher concentrations (2.5 vs 1.5 wt %) produced clearer intersecting structures for both blend fractions (i.e., 20/80 and 50/50). These two observations, in terms of weight fraction and CQ content, indicate the importance of selecting the relative weight fractions of the two components and photoinitiator concentration in the formulation to produce intersecting waveguide structures of better structural quality, which is associated with the phase separating properties, as will be discussed later. Overall, the presence of the intersecting structures in all samples lends itself to the examination of their optical waveguide properties for the transmission of light and their light collection performance in solar cell encapsulants. While the optical microscopy images reveal insight into the formation of the intersecting structure in terms of the polymer blend formulation, they should not be overinterpreted with regard to the dielectric structure of the waveguide lattices. Their optical properties, and the core–cladding structure, are best analyzed through optical transmission studies.

**Figure 3:**
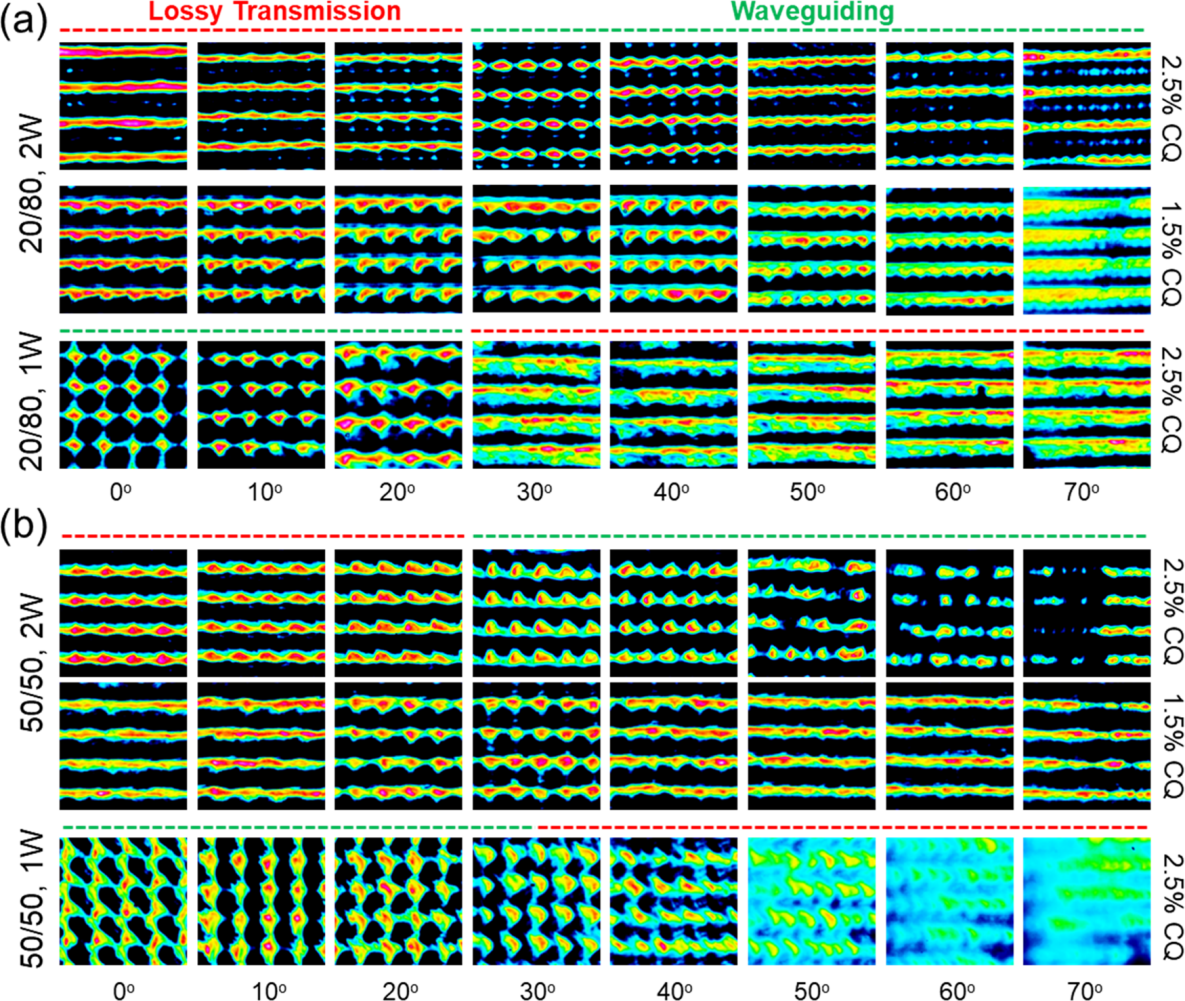
Transverse intensity profiles of transmitted incandescent light (exiting the samples) as a function of angle of incidence (for light entering the samples) for multidirectional waveguide lattices prepared from (a) 20/80 and (b) 50/50 blends with 1.5 and 2.5 wt % CQ and corresponding single vertically aligned waveguide array (for comparison). All samples were irradiated at an optical intensity of 12 mW/cm^2^. Incident angle ranges visually indicative of waveguiding (green) and lossy transmission (red) are shown above the images for each sample.

Figure 3 shows CCD images of the transverse optical intensity profile of transmitted incandescent white light through the intersecting waveguide lattice structures and a single vertically aligned waveguide array (for comparison) as a function of incident angle. The spotted nature of the profiles for specific incident angles indicates transmitted light is predominantly confined to the waveguide cores and exits them to form these localized spot intensities shown in the CCD images. The well-ordered intensity pattern also correlates to an underlying well-formed waveguide array structure.^33^ At incident angles beyond the acceptance ranges, the transmitted light appears to exit the film outside of the waveguide cores and the spot intensities begin to overlap or smear into one another. This indicates a transition to lossy mode transmission, in which a portion of light that enters the waveguides will refract out of it and enter an adjacent waveguide, repeating this process to form a “smeared” intensity pattern. The remaining portion of the light may remain reflected within the waveguide cores, resulting in some resemblance of the spot pattern for incident angles beyond the acceptance range. The portion of light no longer confined to the waveguides (i.e., lossy transmission) increases as the incident angle moves further away from the collection range, where eventually light simply cuts through the waveguides, resulting in transmission profiles appearing as lamellae of high-intensity light. The multidirectional waveguide lattice structure consists of intersecting waveguides on a common plane, and such planes stack in the vertical direction. Hence, beyond the acceptance range, these stacked layers, or slabs, operate more like higher-index layers, relative to the interstices in between the layers, and confine light to this layer, which explains why there is in turn the appearance of “stacked layers” of dark regions.

The transverse intensity profiles of transmission light at the exit of the film show how confinement of optical intensity to the waveguides occurs at greater incident angles (ca. 30–70°) for intersecting waveguide arrays, in accordance with the slant waveguide orientations (25°) rotating their collection range to accept light at greater incident angles (i.e., more oblique, away from the surface normal). At high incident angles, the spots appear to overlap due to their closer proximity when viewing their profiles at such glancing angles. However, the circular-shaped intensity spot profiles are still discernible and would indicate the persistence of waveguided transmission at very large incident angles, as will be further evidenced when calculating their experimental collection angles, as discussed later. The multidirectional waveguide structures produced from 20/80 blends show clear spots from the onset of light collection (∼25°) up to 70°, indicating confinement and emission from their waveguide cores over this very large angular range. In the case of the 50/50 blends, the periodically ordered arrangement of the transmission spots begins to deteriorate, unlike with the 20/80 blends. One explanation could be the decreasing quality of the cylindrical geometry of the waveguide structures produced with the 50/50 blends consequently leading to a loss in the capability to confine light. However, the transmission profiles for moderate incident angles (20–40°) appear to be visually as good as for structures produced from the 20/80 blends. Hence, a more convincing explanation might be that at greater incident angles, light will couple into higher-order modes of the waveguides, which are more sensitive to the quality and uniformity of the waveguide core,^32,41^ and consequently will not be as efficiently confined. In simpler terms, with increasing incident angle, the capability for the waveguide structures to effectively confine collected light becomes more sensitive to the quality of the waveguide structures. In the case of the single-waveguide arrays (0°), produced from either of the weight fractions, collection and waveguided transmission occur at lower incident angles (ca. 0–20°), but this capability is lost at greater incident angles. Importantly, the intersecting waveguide structures prepared from both 20/80 and 50/50 blends rotationally shift the collection capabilities to greater incident angles relative to the control, all be it with different effectiveness. The collection properties for each waveguide array in the structure (i.e., −25 and 25°) showed no difference (see the Supporting Information), and hence the irradiation setup was able to produce structures with truly symmetric optical properties. When comparing the optical transmission between blends prepared with 2.5 and 1.5 wt %, blends prepared with higher CQ concentration confine light more tightly, with their CCD images showing a sharper spot pattern (i.e., smaller size spots) in the regime of waveguided transmission. This is most evident, for example, when examining incident angles of 30 and 40°. Also, in the case of the 40° incident angle, the 50/50 blend prepared at 1.5 wt % CQ produces a smeared CCD pattern, whereas the corresponding 2.5 wt % CQ still shows a spotted pattern. It is also possible that the formation of lamellae for 50/50 blends prepared with 1.5 wt % CQ is also a result of the lower quality of the waveguide ends produced with this formulation, as shown in the corresponding optical microscopy images. These observations point toward 20/80 blends with 2.5 wt % CQ as the formulation that produces, visually, the sharpest, highest-quality transmission patterns over the greatest angular range. Examination of these intensity profiles is suitable for observation of light confinement as a function of incident angle, but not necessarily the total light transmitted, which may differ based on other aspects, such as scattering, absorbance, etc. Hence, while light collection regimes are indicated by the CCD images, examining the solar cell response will indicate the combined performance of the efficient collection, transmission, and finally conversion when the polymer structures are employed as encapsulants, which will be discussed later.

The observed collection intervals (and their range) are directly correlated to the refractive indices of the waveguide cores and cladding (i.e., their index difference, Δ*n*), which is in turn related to the relative volume fractions of the blends, and thus can be used to infer degrees of phase separation in the core and cladding. To model the observed acceptance angle range for the waveguides in a slant orientation, we first correlate the boundaries of the acceptance range to the refractive index difference (Δ*n*) between the polymer waveguide cores and their surroundings (consider as the cladding). This refractive index difference is expressed in terms of the refractive index of the core and cladding, both of which depend on the volume fraction of each polymer in their respective regions. To begin, we define the bulk volume fraction (φ_bulk_) as that of the free-radical polymerizing component, NOA65 in our case, which is fixed by the initial formulation (50 or 80%), and may be expressed as:
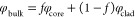
1where *f* is the volume fraction occupied by the waveguide cores, and *f*_core_ and *f*_cladd_ are the volume fraction of the high-index polymer (NOA65) in the waveguide core and surrounding medium (i.e., the cladding of the waveguide), respectively. With the densities of the polymers being ∼1 g/cm^2^, the volume fractions are nominally the same as the weight fractions expressed herein. During phase separation, *f*_core_ is expected to increase owing to the phase separation (i.e., expulsion) of PDMS into the surroundings and concurrent diffusion of NOA65 (to preserve mass balance) into the polymerizing waveguide cores. Likewise, *f*_cladd_ will decrease as NOA65 is lost to the polymerizing cores and PDMS content increases (via gaining PDMS expelled from the polymerizing regions). The volume fraction occupied by the waveguides based on a model of two intersecting cylinders along a common plane within a rectangular unit cell is
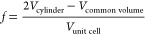
2where *V*_cylinder_ is the volume of a slanted cylinder in a rectangular prism cell, *V*_commonvolume_ is the common volume or volume of overlap of the two cylinders with the same radius,^45^ which must be subtracted so as not doubly count cylinder occupancy, and *V*_cell_ is the volume of the rectangular unit cell. Equation 2 expands to be expressed in terms of the waveguide cylindrical radius (*r*), mask aperture spacing (*S*), which defines the square base of the unit cell, and the orientation angle of the waveguide (θ) relative to the surface normal
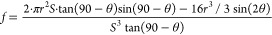
3The theoretical fraction dictated by the mask (*r* = 20, *S* = 200, and θ = 25°), i.e., if the waveguides have the same diameter as the mask apertures, is approximately 5.4%. Measurements of the waveguides show their diameter is approximately 46 μm, which gives a volume fraction of 7.0%. Next, the volume-averaged refractive indices of the waveguide cores and surroundings are
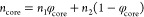
4
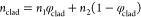
5where *n*_1_ and *n*_2_ are the refractive indices of NOA65 and PDMS, respectively. The boundaries for the angular acceptance range (θ_a_) for a slant waveguide of angle θ_w_ at the air–core interface can be determined to be
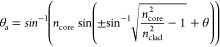
6Briefly, the approach to derive eq 6 is to first calculate the boundaries of the acceptances angle of a core immersed in the cladding medium, rotate those bounds by the waveguide orientation angle (i.e., addition of θ), and then determine their respective values considering refraction at the air–core interface. Hence, given the collection angles determined experimentally, the angle of orientation of the waveguides, and volume fraction of the waveguides in the array, one can predict the polymer volume fractions in the waveguide and surrounding regions. In more detail, the volume fraction of the core (*f*_core_) can be used as fit parameter via eqs 1–6 to regress the experimental boundaries of the acceptance range. The CCD images of transmission intensity would indicate an approximate lower bound to the acceptance range of ∼25 to ∼30° and up to approximately 70°, for 20/80 and 50/50 blends, respectively. In the case of the lower bound, this is determined by the angle at which there is approximately the first instance of distinct (i.e., nonoverlapping) spots in the optical intensity profiles. Since the experiment was limited to observing incident angles up to 70°, and the spot patterns appear to persist beyond this angle, the upper bound of the acceptance range is uncertain and could be higher than 70°. Hence, the lower bound angle of the acceptance cone was used as the angle to fit. For the 20/80 blend, using a lower bound of 25° as input, the refractive indices of the core and the cladding are determined to be 1.627 and 1.603 (Δ*n* = 0.024), respectively. This would indicate that the upper bound to the acceptance range is approximately 85°. The volume fractions of NOA65 that account for these refractive index values for the waveguide core and surroundings would be 89.4 and 79.3%, respectively. Hence, the waveguide cores become enriched from the initial 80% of the bulk mixture (prior to irradiation) by 9.4%, and the surrounding mixture becomes diluted by 0.7%. This low dilution in the surrounding regions is a result of the small volume fraction of the growing waveguides in the medium from which PDMS may be expelled. In other words, the volume of PDMS expelled from the regions of irradiation is so small that it leads to a very small change in the polymer blend composition in the nonirradiated regions. Likewise, for 50/50 blends using a lower bound of ∼30° as input, the refractive index of the core and the cladding are determined to be approximately 1.54 and 1.53 (Δ*n* = 0.01), respectively, indicating an ∼53.7% NOA65-rich core and 49.7% NOA65 in the surroundings. Once again, the waveguide is enriched, less so than with the 20/80 blend, and the surroundings are slightly diluted. In terms of the refractive index profile, therefore, the waveguide structures consist of cores enriched with NOA65 that are embedded in a bulk medium that is only slightly lower in refractive index than the original bulk mixture. Nevertheless, the refractive index difference is sufficient to yield wide acceptance ranges for light collection. As a comparison, the acceptance range for the single vertical waveguide array, assuming the maximum collection angle is ∼20° (observed in the CCD images) yields a 95% NOA65 volume fraction in the cores (*f* = 3.5%) and 79% in the surroundings. This is in good agreement with measurements of degrees of phase separation in previous work.^29,40^ As the collection ranges were not significantly different between the two CQ concentrations, the collection angles could not be used to back-calculate any difference in the degrees of phase separation of the blends when using different CQ amounts. It is possible that differences observed in subsequent solar cell performance (to be discussed in the next section) are a result more so of internal waveguide morphology. The varied collection ranges to the extent at which they can be used to infer phase separation simply indicates an evident difference when preparing intersecting waveguide structures from blends with different fractions of the polymer components. The similar CCD intensity patterns as a function of incident angle observed for the other waveguide in the array (see the Supporting Information) indicate that it would also have the same collection ranges and degrees of separation determined herein.

One explanation for the higher degree of phase separation in the single-waveguide array over the multiwaveguide array may be the greater overall exposure of the medium for the latter, resulting in greater degrees of cure, which can slow down the dynamics of phase separation. However, for the concentration of NOA65 to reach 95% in the multidirectional waveguide lattice structures, the boundaries of the acceptance range should be ca. 20–90° and ca. 14–90° for 20/80 and 50/50 blends, respectively, notably decreasing the lower bound of the acceptance range. This additional enhancement to the lower bound of the acceptance range is not necessary: Light within the 0–20° can be sufficiently collected and converted in the solar cell; rather, wide-angle capture is the important operational feature of the slanted waveguides. Likewise, beyond incident angles of 70°, significant Fresnel reflection at the air–polymer interface plays a greater role in the loss of light than any shading or loss effects at the solar cell surface. Hence, the boundaries of the acceptance range as achieved with the structures herein are sufficient, compared to what might possibly be achieved with greater degrees of phase separation, and certainly sufficient to demonstrate the concept of enhancing wide-angle light collection compared to single vertically aligned waveguide arrays, as indicated by the following discussion on solar cell performance. Finally, it is important to note that discerning collection ranges for the boundaries of the acceptance range from the transmission intensity profiles are only a visual approximation. There is no sharp transition between the regimes of waveguiding and lossy transmission, but rather with an increased angle (away from the acceptance range), a greater portion of light will be able to pass through the waveguides. Hence, the selection of 25 and 30° as the lower bound for the 20/80 blend was a middle number from the observed approximate transition from what visually appeared to be the formation of distinct spots. Likewise, the selection of 30° for the 50/50 blend was an approximate for the transition to the profile possessing a strong spotted pattern.

**Figure 4:**
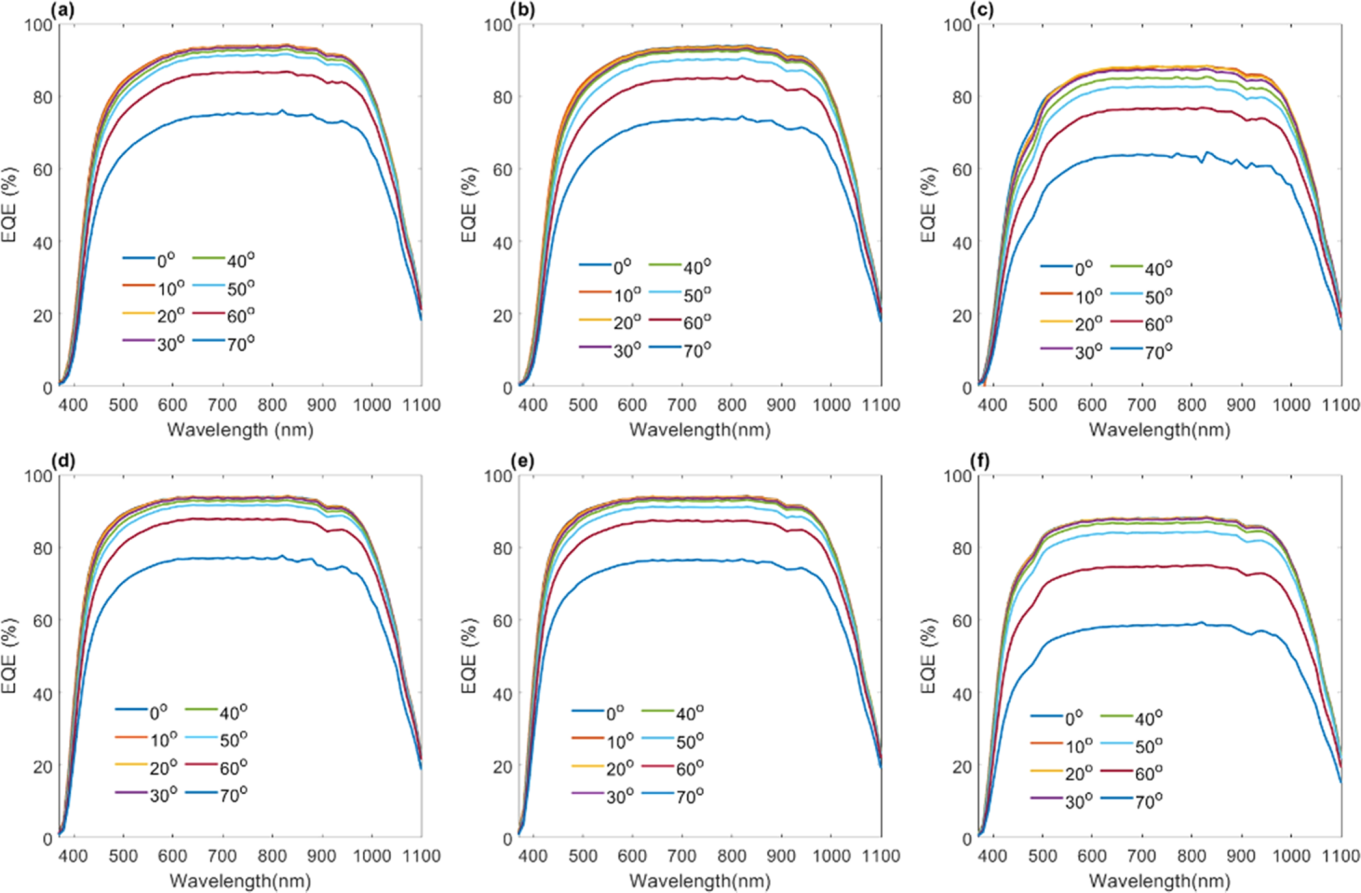
Angle-resolved EQE spectra of solar cells encapsulated with waveguide structures. (a, b) Intersecting waveguide structures produced from 50/50 polymer blends with 2.5 and 1.5 wt % CQ, respectively. (c) Single vertical waveguide array produced from 50/50 polymer blends with 2.5 wt % CQ. (d, e) Intersecting waveguide structures produced from 20/80 polymer blends with 2.5 and 1.5 wt % CQ, respectively. (f) Single vertical waveguide array produced from 20/80 polymer blends with 2.5 wt % CQ. Single vertical waveguide arrays shown in (c) and (f) are used for comparison to the intersecting waveguide structures produced with the same polymer blend fractions.

Figure 4 shows angle-resolved EQE spectra for the multiwaveguide arrays and single vertical waveguide arrays for both 50/50 and 20/80 weight fractions. The spectral curves naturally drop with increased incident angle both because of unavoidable Fresnel reflection at the air–polymer interface and the shading losses from the silicon solar cell top contacts.^13,20^ We have already shown that the single vertical waveguide array shows a lesser drop than a uniform coating.^20^Figure 4 now shows that the multidirectional waveguide lattice structures enable the EQE to drop less, evident in the most responsive region of the solar cells (i.e., EQE plateaus at ca. 600–900 nm), compared to the single vertically aligned waveguide array. This enhancement is observed especially at higher incident angles, 50–70° down to an incident angle of 40° showing spectra positioned among those for smaller incident angles. These EQE curves showing higher values clearly indicate an enhancement in the incident angle-dependent conversion efficiency. The multiwaveguide lattice structures produced with 1.5 and 2.5 wt % CQ both visually show similar angle-dependent EQE responses, both of which are greater than that achieved with a single vertically aligned waveguide array.

**Figure 5:**
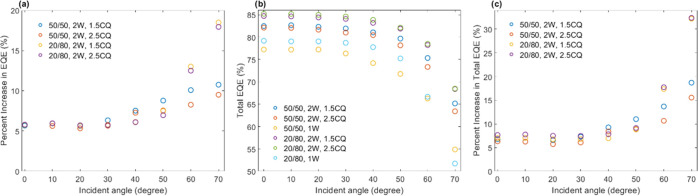
Quantitative analysis of encapsulated solar cell performance. (a) Percent increase in EQE (examined at ∼770 nm in the spectra) of intersecting waveguide encapsulant structures (produced with two different CQ concentrations) with respect to their single vertical waveguide array as a function of angle of incidence; 770 nm was selected as the region of approximate maximal response of the solar cells. (b) Total EQE as a function of incident angle for both intersecting waveguide lattices and singe vertical waveguide array encapsulants. (c) Percent increase in total EQE for intersecting waveguide structures with respect to single vertical waveguide arrays.

To more closely and quantitatively describe the enhancements, Figure 5a shows plots the percent increase in the maximum EQE value (found at ∼770 nm within the spectral plateau) for the EQE curves in Figure 4 for the multiwaveguide arrays relative to the single-waveguide array. Indeed, all multiwaveguide arrays show enhancements in conversion efficiency, specifically an increasing enhancement (i.e., percent increase) with increased incident angle. Structures produced from 20/80 blends show greater enhancement than those produced from the 50/50 blends. Likewise, structures produced with 2.5 wt % CQ perform better than those produced with 1.5 wt % CQ. We further calculated the total EQE across the entire response spectrum as a function of incident angle, as shown in Figure 5b. Once again, the 20/80 blend outperforms the 50/50, but the difference between the use of 1.5 and 2.5 wt % CQ in the formulations on the total EQE values is not clear. Specifically, structures from 20/80 blends with 2.5 wt % CQ yield a marginally greater total EQE compared with using 1.5 wt % CQ, which diminishes with increased angle of incidence. On the other hand, for 50/50 blends, using 1.5 wt % shows a slightly greater total EQE compared with 2.5 wt % CQ, which also diminishes with a greater incident angle. The difference between the enhancements revealed by examining the EQE plateau and the total EQE (Figure 5a,b) may be a result of the selection of the middle of the EQE curve plateaus as a more subjective indication of enhancement, with the total EQE over the entire response spectrum being more representative of performance. Hence, the EQE curves represent more of an indication of the presence of enhancement, but the total EQE should be used for a more accurate assessment. Hence, Figure 5c shows the percent increase in total EQE as a function of incident angle for the multiwaveguide structures relative to the single vertically aligned waveguide array. The plots show that structures produced from both 20/80 and 50/50 blends with either 1.5 or 2.5 wt % CQ provide enhancement in the total EQE. Interestingly, all structures show an approximate constant improvement of ca. 6–7% for low incident angles of 0–30°. The percent increase in the total EQE then begins to rise after 30°, which is within the calculated acceptance cone of the slanted waveguides (>25°) of the multiwaveguide structure and beyond that for the single vertically aligned waveguide array (<∼20°). This results in the steady monotonic increase in the gained total EQE as collection effectively persists for the multidirectional waveguides but drops for the single-waveguide array. The combined results of the transverse intensity profiles and the EQE response as well as the predicted collection ranges of the structures provide compelling evidence that the multiwaveguide array structures provide an enhancement in wide-angle energy conversion by virtue of the structure-light collection properties.

It is interesting to note that all intersecting waveguide structures provide a constant, baseline improvement to the total EQE over the single vertically aligned waveguide array for the low incident angle range of 0–20°. One explanation is that transmitted light beyond the waveguide acceptance range still interacts with the waveguide structures, cutting through them at low incident angles beyond their acceptance range, but still possibly being collected to some extent (lossy transmission) or even possibly scattered by the internal waveguide morphology. The confinement into the high-refractive-index planes of intersection of the waveguides is already demonstrated at low angles in Figure 3. Hence, any interaction (e.g., scattering or collection as lossy modes) that possibly enables light to reach the solar cell surface at a non-normal incidence can aid in total light collected. This is a well-known phenomenon employed in solar cells: scattering light internally away from normal incidence, which is often achieved by surface texturing the solar cell surface. When light is redirected away from the normal incident pathway, it is more likely to be confined within the encapsulant because the light will bounce within the encapsulant at angles outside of the “loss cone”. The loss cone is a range of incident angles of light inside the encapsulant whereby a portion of which will be lost by refraction back through the air–encapsulant interface. Hence, in addition to its light collecting properties when light is within the acceptance cone, beyond the acceptance cone, the structures appear to provide the capability to still collect, confine, and possibly scatter light, thereby providing interactions and enabling transmission properties of light to be advantageously altered toward improving total energy conversion efficiency. Such effects have been revealed in previous simulation studies.^20,24^

**Figure 6:**
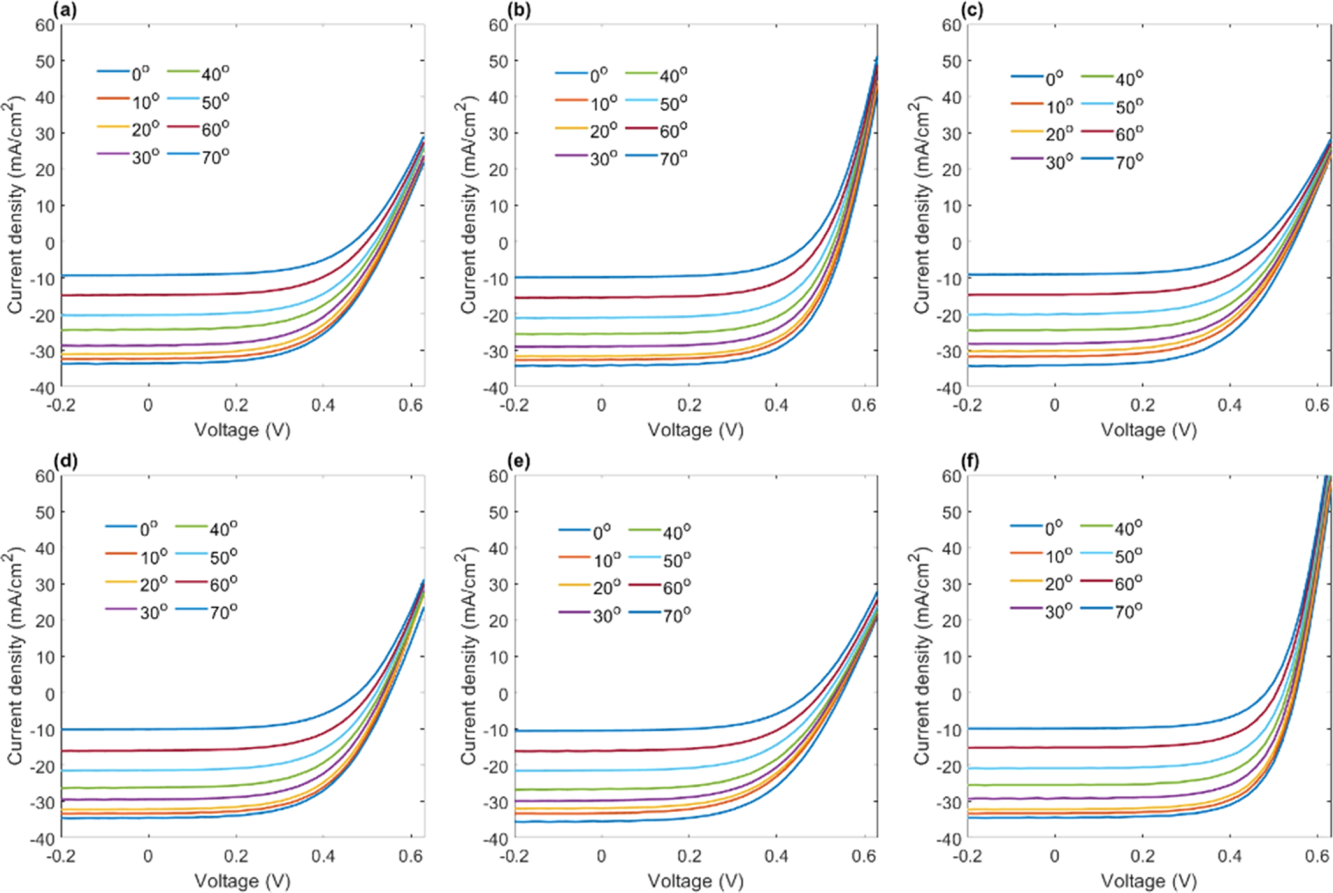
Angle-resolved current density–voltage (*J*–*V*) curves of solar cells encapsulated with waveguide structures. (a, b) Intersecting waveguide structures produced from 50/50 polymer blends with 2.5 and 1.5 wt % CQ, respectively. (c) Single vertical waveguide array produced from 50/50 polymer blends with 2.5 wt % CQ. (d, e) Intersecting waveguide structures produced from 20/80 polymer blends with 2.5 and 1.5 wt % CQ, respectively. (f) Single vertical waveguide array produced from 20/80 polymer blends with 2.5 wt % CQ. Single vertical waveguide arrays shown in (c) and (f) are used for comparison to the intersecting waveguide structures produced with the same polymer blend fractions.

**Figure 7:**
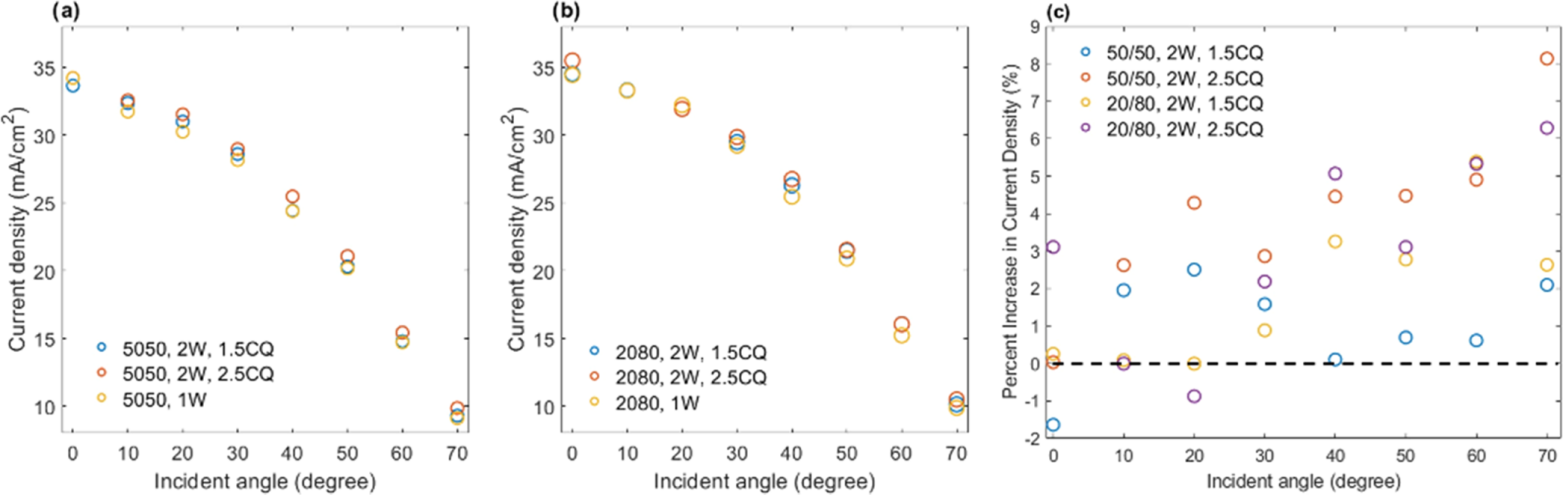
Analysis of electrical output in encapsulated solar cells. (a, b) Short-circuit current density (*J*_SC_) as a function of incident angle for encapsulants produced from (a) 50/50 and (b) 20/80 polymer blends. (c) Percent increase in *J*_SC_ of intersecting waveguides with respect to a single vertical waveguide array as a function of incident angle. The dashed line aids to identify positive and negative performance changes when employing intersecting multiwaveguide structures.

Figure 6 shows the current density–voltage (*J*–*V*) curves of the encapsulated solar cells under AM 1.5 G solar irradiation. The curves show the natural decrease in the current output, as indicated by the drop in the short-circuit current density (*J*_SC_, at 0 V). However, the curves reveal small increases in the short-circuit current density of the multidirectional waveguide arrays vs the corresponding single vertically aligned waveguide arrays. To show this enhancement more clearly, plots of *J*_SC_ vs incident angle (Figure 7a,b) indicate that solar cells with multidirectional waveguide encapsulations provide a small but consistent increase in the current density relative to a single vertically aligned waveguide array, which becomes more pronounced with increased incident angle. The average increase in the short-circuit current density of the entire incident angular range was 0.36 and 0.60 mA/cm^2^ for 20/80 blends with 1.5 and 2.5 wt % CQ, respectively, and likewise, the average increase in the short-circuit current density was 0.22 and 0.80 mA/cm^2^ for 50/50 blends with 1.5 and 2.5 wt % CQ, respectively. These nominal increases could also be expressed as average percent increases: 1.91 and 3.03% for 20/80 blends with 1.5 and 2.5 wt % CQ, respectively, and 1.00 and 3.98% for 50/50 blends with 1.5 and 2.5 wt % CQ, respectively. If only the ultrawide angles (50–70°) are considered, the increases in short-circuit current density are 0.55 (3.6%) and 0.69 (4.91%) for 20/80 blends and 0.14 (1.14%) and 0.79 (5.84%) for 50/50 blends, both for 1.5 and 2.5 wt % CQ, respectively. The percent increases in the short-circuit current density as a function of incident angle are plotted in Figure 7c. While the values visually appear to fluctuate, there is indeed a positive trend to the increase in the short-circuit current density with increased incident angle. The trends once again confirm that formulations prepared with 2.5 wt % CQ as well as those from 20/80 blends provided the greatest increase in the current output; yet comparatively, all multidirectional lattices provide an overall improvement compared to a single-waveguide array both for moderate as well as very wide incident angles. It is notable that current output is enhanced even below the lower bound of the acceptance range, confirming that light can still positively interact with the multidirectional waveguide lattice structure. It is also notable that there are instances of no improvement or loss in performance in the percent increase of *J*_SC_ of some encapsulant structures, such as 50/50 with 1.5 wt % CQ, which would indicate the importance of selecting the better weight fraction (and CQ concentration) to improve the encapsulant properties, to improve electrical performance.

It is important to note that both waveguide arrays in the structure (i.e., ±25° orientations) show similar performance in terms of solar cell efficiency enhancement and the electrical output (see the Supporting Information). Hence, symmetric structures with symmetric optical properties and solar cell performance indicate that ultrawide-angle light capture (−70 to 70°) can be achieved through the photopolymerization approach employing two LED sources.

## Discussion

The combined characterization of the structure, light acceptance ranges, solar cell conversion efficiency, and current output provides insights into the overall processing–structure–property–performance relationship, for which the photopolymerization and phase separation of the blend are central. The formation of the waveguides entails first the propagation of polymerized fiber/rod-shaped structure across the medium, then subsequent polymerization and increased molecular weight, which induces the blend in the irradiated region to become thermodynamically unstable and to phase-separate.^32^ This phase separation occurs while the molecular weights of the components continue to grow, which increases the system viscosity and elasticity, thereby reducing the diffusivity of the blend components. The kinetic and dynamic balance between photopolymerization and phase separation allows well-shaped cylindrical phases to form, which possess high degrees of phase separation, in other words, polymer blends with well-defined and well-patterned structures and binary phase morphologies. For the dynamics of phase separation to proceed quicker than it takes for the system to increase molecular weight, to reduce those dynamics, it is possible that the phase-separated morphology can deviate from the cylindrical pattern dictated by the optical beams. Likewise, if the polymerization rate is too high, the sample can solidify before any significant phase separation is possible.

The results indicate that the relative weight fractions of the blend (i.e., 20/80 vs 50/50) play a greater role in the quality of the cylindrical waveguide geometry. This is especially the case when considering the depth dependence, where the 50/50 blends show waveguide cores that diverge or “splay” more so at their ends. This is associated with the lag in the polymerization rate and molecular weight increase at greater depths in the blend,^32^ allowing phase separation to proceed more than at lesser depths, and possibly, as in this case, causing the blend morphology to deviate from the cylindrical geometry. However, the greater concentration of the free-radical polymer (NOA65) in the 20/80 blends allows the entire length of the waveguide cores to retain a uniform cylindrical shape. Furthermore, the 20/80 blends have a more direct thermodynamic pathway toward phase separation (i.e., 80 100), as the blend will favor increasing NOA65 and expelling PDMS. This would also enable the system to more quickly and easily achieve a greater refractive index, which aids in keeping light confined to the irradiated region, to enable cylindrical cores to grow with a more uniform width over its length within the polymer medium. It is also possible that the diverging morphology in the 50/50 blend captures an imprint of the diverging nature of the light at very large depths. Greater CQ results in greater photoinitiation and polymerization rate, which can increase the molecular weight faster, enabling a greater cross-linking density, whereby the waveguide core’s integrity in terms of cylindrical shape and morphology is maintained during phase separation. It can also in turn cause the refractive index difference of the growing waveguide to rise quicker, which aids in confining light (i.e., less divergence at the ends). This is more so the case with 20/80 blends, with its greater NOA65 content forming robust waveguide cores and expelling the PDMS without losing the quality of its structure. This is in accordance with previous work which revealed that symmetric blends (i.e., 50/50) have a tendency to undergo phase evolution unmitigated by the optical beams and that weight fractions with higher asymmetry in the relative weight fractions mitigate phase evolution and are more suitable for capturing the intended pattern of the optical beams.^32,41^ Greater polymerization degrees drive phase separation and increase the refractive index difference, which provides better collection and waveguiding of light over a larger acceptance range. The observation of more asymmetric polymer blends and greater polymerization rates producing better quality waveguides provides experimental verification of our theoretical predictions from our Multiphysics framework on structure and morphology formation.^44^ It is also possible that there are variations in the degree of polymerization between regions irradiated by a single beam and regions in which two beams overlap, as well as the dark regions, as indicated in our simulation work.^44^ Detailed investigation of the spatially resolved conversion in different regions of the structure is currently underway and will be reported in the future.

The model between collection angle, blend volume fraction, and refractive index difference shows that the cores show a significant degree of phase separation but not complete phase separation and that the surroundings are only mildly changed in terms of blend composition and refractive index. Hence, the structure is best described as one in which there is an NOA65-rich core, and a mildly richer PDMS cladding. This contrasts with the 95% NOA65 observed for vertically aligned single-waveguide arrays, most likely achieved by the lower and more isolated exposure by a single array of optical beams allowing more time and mobility to achieve a greater degree of phase separation, in contrast to herein where there is double the total exposure using two LEDs. Their slant angles also give them a greater path length, thus exposing more of the medium to irradiation, which can increase the photopolymerization rate and thoroughness of the cure.

While the volume fraction of the NOA65 has increased (and PDMS decreased) in the waveguide cores owing to the phase separation, the lack of complete phase separation implies that some PDMS remains in the cores. Also, the volume fraction in the surrounding medium is only slightly lower than the bulk volume fraction of 80%, owing to the very low volume fraction of the PDMS in the cores only minutely diluting the NOA65 in the surroundings. This indicates a correlation between waveguide density in the array (not explored herein, but to make the point) and potential refractive index difference: the more waveguides form in a volume (i.e., waveguide density), the greater the volume of PDMS that can be expelled into the surroundings, allowing greater dilution and greater achievable refractive index differences between the core and cladding. However, more dense arrays will require mask patterns with density aperture patterns, which increases the overall exposure of the medium during photopolymerization (i.e., denser arrays of optical beams), with the unintended result being faster curing kinetics quickly arresting the very phase separation dynamics needed to evolve the high-refractive-index difference to yield wide acceptance ranges. This is most likely why the collection angles do not vary significantly between sparse or density waveguide arrays^29^ because any potentially greater collection window from a greater refractive index from denser waveguides is inhibited by the greater light exposure and arrested phase separation dynamics owing to greater exposure hindering their formation. Nevertheless, the dependence of the waveguide structure and collection properties on waveguide size and density is the subject of continued work that will be reported in the future.

Note that the degree of separation does not imply that the waveguides themselves are uniform in the blend distribution throughout the cross section and length of the waveguide cores. It is certainly possible that phase separation has occurred internally and that the refractive index properties of the cores are related more so to a volume average of the smaller phases. The presence of “streaks” or “striations” along the lengths of the waveguides might be indicative of internal morphology in the waveguide cores. This may also be the case, more so, for the 50/50 blends, which can produce more random phase separation or a coarser morphology in the waveguide cores, which, while not significantly affecting the apparent collection angles, can affect the energy conversion and electrical output. Nevertheless, modulations in light transmission and improvements in solar cell performance are still observed. This indicates that it is not necessary to obtain “perfect” uniformity in the waveguides to significantly modulate light transmission, or, on the other hand, this may indicate potential opportunities for improved performance if the processing conditions can produce a more uniform core–cladding geometry. Simulations indicate that a high polymerization rate is favorable for achieving well-defined intersecting structures.^44^ Hence, increased photoinitiator or light intensity may be considered, so long as they are not too high to rapidly cure the blend such that phase separation is arrested or significantly inhibited. We produced our structures at the maximum intensity of our LED sources, and a higher CQ content might not be desirable owing to the possible remnant CQ absorbing in the blue region of solar radiation. One approach going forward would be to employ UV LEDs for the combination of rapid curing and use of an efficient UV photoinitiator, which may provide higher polymerization rates and non-visible-light absorbance, to enable better quality waveguides to be produced, and such studies are currently underway. Combining these multidirectional waveguide structures with some type of micro-lens array placed on top of it might aid with collection at extreme incident angles (i.e., 60–70°) by providing stronger re-steering into the film at smaller angles, as an approach to reduce losses from Fresnel reflection at the planar air–polymer interface, but this additional advance to encapsulant architecture is beyond the scope of this work.

Overall, through the selection of appropriate relative weight fractions of the blend and the photoinitiator concentration, high-quality waveguides may be produced, with well characterized and modeled acceptance ranges that correlate to their capability to enable greater wide-angle light collection, conversion, and electrical output when employed as solar cell encapsulants. It is important to note that improvements in the current density even on the order of ∼1 mA/cm^2^ is significant, especially when considering the total area of a solar cell and solar panel. For example, as of 2020, current individual solar cells are 21 cm × 21 cm, and panels may contain either 60 or 72 cells. Hence, a nominal increase of 0.6 mA/cm^2^ achieved with an encapsulant produced from 20/80 blends and 2.5 wt % CQ, for example, translates to an increase in current output of approximately 15.9 and 19.1 A per 60- and 72-cell panel, respectively. These output currents would also scale with the number of panels in a solar farm. Solar panels placed in nonoptimized locations (building or residential roofs) would also see a benefit from these enhanced collection properties. Long-term rooftop studies of collection, conversion, and electrical output of encapsulated solar cells over the course of the day and over seasons are currently planned and will be reported in the future.

## Conclusions

We have produced multiple intersecting waveguide array structures in photopolymerizable polymer blends to enable wide-angle light capture and waveguide-based light transmission in thin polymer films as novel encapsulation materials for solar cells. The structures demonstrate the capability to accept very high incident angle radiation, enabling their transmission and incidence on the solar cell surface at angles smaller than those dictated by natural refraction. Thereby, more direct incident radiation mitigates front-contact losses, thereby sustaining energy conversion and current output over a larger angular range, and especially at wide incident angles up to 70°. The structure and performance of the multidirectional waveguide array structures are correlated to the relative weight fractions of the polymer blend components and free-radical photoinitiator concentration: greater fractions of high-refractive-index polymer and photoinitiator correlate to better waveguide structures and improved light collection, transmission, energy conversion, and solar cell output. These blend parameters allow for better evolution of the medium into higher-quality core–cladding architectures for the collection, confinement, and waveguide-based transmission of light. Improved performance is also observed for small incident angles, beyond the acceptance range, owing to increased interactions of the waveguide structures with light. This work demonstrates the capability to design advanced multiple-waveguide architectures in thin films with novel transmission properties toward improved solar energy collection and conversion in solar cells. This capability can extend and sustain solar cell performance over the course of the day and across seasons, as the incident angle of solar irradiation varies, enabling greater total power output, which can increase the capability of clean, renewable solar energy to contribute power to the grid.

## Supporting Information Available

Optical characterization and solar cell performance for other waveguide sets and polymer blend conditions (PDF)
