# Supplementary material for: Multidirectional Polymer Waveguide Lattices for Enhanced Ultrawide-Angle Light Capture in Silicon Solar Cells
Source: ACS Appl Energy Mater. 2022 Jul 22;5(8):9980–93. doi: 10.1021/acsaem.2c01630 (PMC9400022; doi:10.1021/acsaem.2c01630)
Supplement: Supplementary file 1 — ae2c01630_si_001.pdf [file ae2c01630_si_001.pdf]

SUPPORTING INFORMATION

Multidirectional Polymer Waveguide Lattices for Enhanced Ultrawide-Angle Light Capture in Silicon Solar Cells

Nannan Ding,[1] Ian D. Hosein[1]*

1. Syracuse University, Department of Biomedical and Chemical Engineering, Syracuse, NY, 13244

* Corresponding Author: idhosein@syr.edu

Examining the other waveguide array to indicate symmetric properties and performance.

The binary blends consisting of NOA65 and PDMS were irradiated with two symmetric optical beams and each array of beams writes its respective array of waveguides into the medium. Namely, two arrays of optical beam write two arrays of waveguides, which are symmetric and theoretically possess same properties, such as waveguide optical properties, same angle with respect to the surface normal, and similar polymer quality.

Herein, solar cell performance from the other array in the structures shown in the manuscript (i.e., oriented at -25°, produced from the other LED in the photopolymerization setup) is presented to demonstrate that fabricated encapsulant possesses same properties for both arrays of waveguides. Comparison of total EQE and short circuit current from two arrays of WG made from their corresponding mixture confirms that each array of WG provides a comparative level of performance within the encapsulants. In conclusion, the intersecting WGs in encapsulants successfully achieved great enhancement in solar cell performance not only by ultra-wide collection window (i.e., -70° – 70°).

Corresponding Optical, Efficiency, and Performance Data

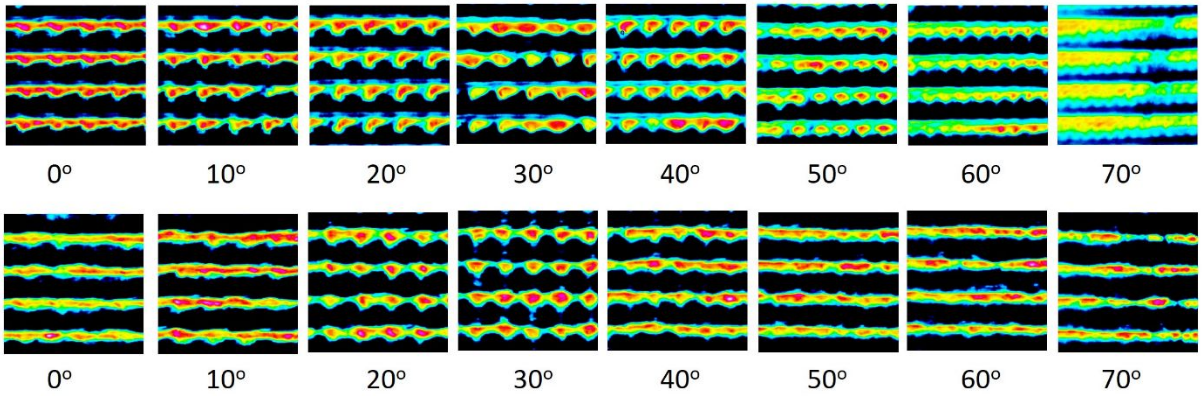

Figure S1. Exemplary CCD images of the transverse optical intensity pattern from the -25° oriented waveguide array from (top) 20/80 blends and (bottom) 50/50 blends.

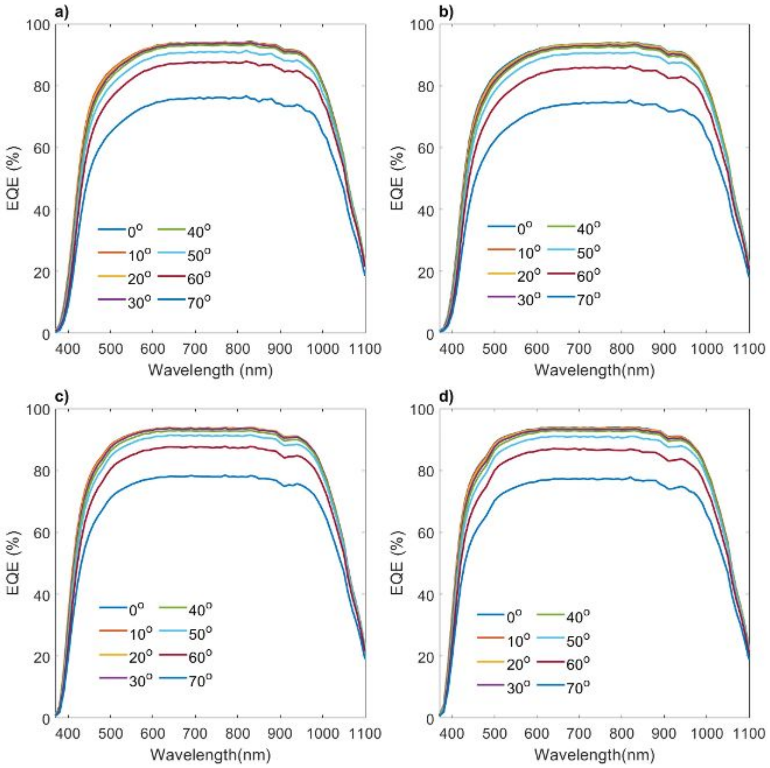

Figure S2. EQE spectrum of coatings encapsulated with two arrays of intersecting waveguides. All four spectra are generated from corresponding -25° oriented arrays (i.e., other waveguide array) in samples presented in the manuscript. a) and b) show 50/50 blends with 2.5 wt% and 1.5 wt% of CQ, respectively. Similarly, c) and d) show 20/80 blends with 2.5 wt% and 1.5 wt% of CQ, respectively.

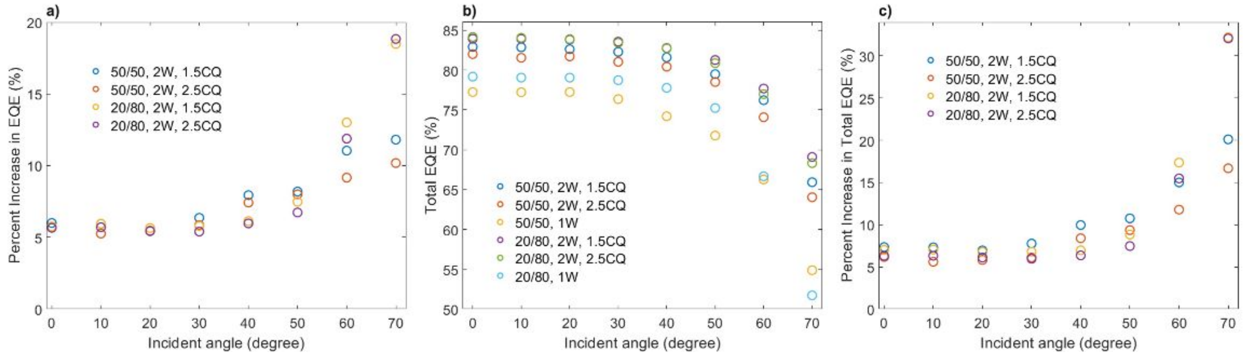

Figure S3. EQE performance. a) Enhancement in EQE of four encapsulants as compared to their respective vertically aligned single waveguide array. b) Plots the total EQE of waveguides over the range of incidence angle. c) Increase in total EQE for all samples explored relative to a single vertically aligned waveguide array, as function of angle of incidence.

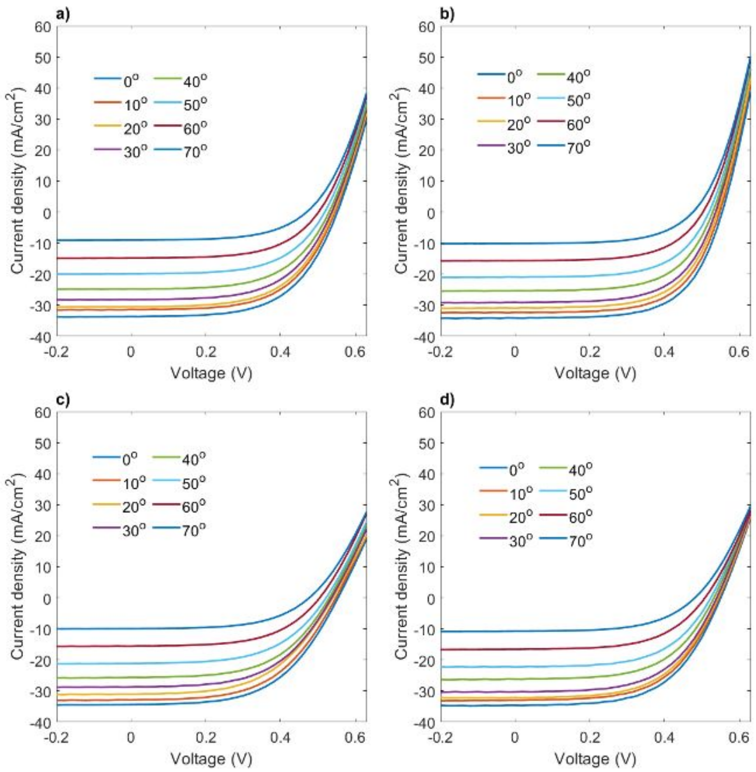

Figure S4. Current Density-Voltage (J-V) curves for all encapsulants comprising of 2WG. a) and b) Polymer blends 50/50 and 2.5 wt% CQ and 1.5 wt% CQ, respectively. c) and d) corresponds to 2.5 wt% and 1.5 wt% of 20/80 mixture.

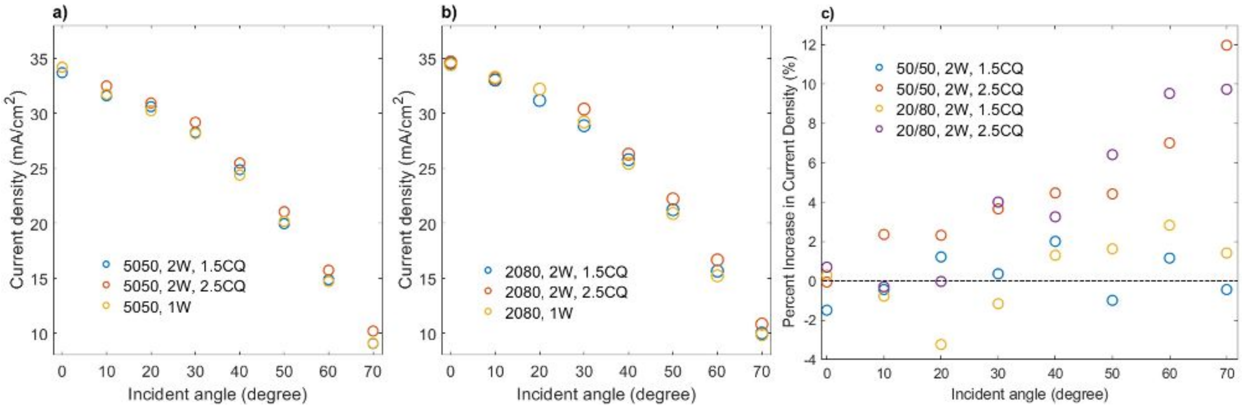

Figure S5. a) and b) show short circuit current over the range of angle of incidence for 50/50 of all samples and 20/80 mixtures, respectively. c) Relative increase in J_{sc} over their respective single WG as a function of incident angle (0° to 70°).
